# Rhizomic learning: How environmental non-governmental organizations (ENGOs) acquire and assemble knowledge

**DOI:** 10.1177/0306312720908343

**Published:** 2020-02-20

**Authors:** Trine E Unander, Knut H Sørensen

**Affiliations:** Department of Interdisciplinary Studies of Culture, Norwegian University of Science and Technology, Norway

**Keywords:** environmental non-governmental organizations, knowledge acquisition, mediation, rhizomic learning

## Abstract

It has been a common assumption that the knowledge practices of environmental organisations (ENGOs) is largely based on interaction with environmental research. Implied in such assumptions is the idea that ENGOs are so-called boundary organizations brokering knowledge between science and environmental policy decision-making. In this article, we challenge this belief. Through interviews, we have investigated the practices of ENGO employees as they acquire and assemble knowledge they need in their involvement with environmental policymakers. From their accounts, these ENGOs are not boundary organizations. Science is important but such knowledge was usually acquired indirectly and appeared to be seen as ubiquitous in the environmental policy community. We found that the knowledge practices were based on what we call rhizomic learning. We introduce this concept to highlight the complexity, opacity and non-linearity of the ways in which ENGO actors acquire and assemble environmental knowledge. We found that this rhizomic learning is characterized by five main features: 1) diversity of sources and the importance of networks, 2) pragmatism, 3) opacity of the process, 4) community among involved actors, and 5) mediation. ENGO actors expected that their capacity for rhizomic learning – not the least the purposeful mediation and assembly of knowledge from a multitude of sources – would make them appear to policymakers as competent, relevant and reliable.

## Introduction

To environmental non-governmental organizations (ENGOs), environmental knowledge is of strategic importance, not the least when they try to influence policy development (e.g., Eden et al., 2006; Jamison, 2011; Yearley, 2005). Such observations raise questions about how ENGO actors acquire and assemble environmental knowledge when working in a context of increasing professional environmentalism and institutionalization (Berny, 2018; Jamison, 2001; Seippel, 2001). To provide answers to such queries, this paper analyzes their learning practices based on interviews with leaders and employees of eight main ENGOs in Norway. The first author interviewed fifteen people for between one and two hours each; the interviews were audiotaped and fully transcribed. She also interviewed fifteen government employees working with environmental, climate and energy issues, and seven politicians from six different parties who had been members of the Parliament’s Standing Committee on Energy and the Environment from 2009 to 2013, but these interviews are only used as a backdrop in this article.

To create a trustful and open exchange, we promised to anonymize the interviewees. This was also explicitly requested by several of them. This means that their organizational affiliation is not disclosed, so we do not analyze differences between the knowledge assembling practices of the ENGOs. For the purpose of the paper, these differences were small and did not add much to the general overview. The selected ENGOs varied in size and with respect to organizational features. Three of the ENGOs were member-based, and two were Norwegian nodes of international organizations. The sources of income included membership dues, private donations, governmental support, support from industry and contracts.^1^ None of the organizations depended on only one source of income. Economic resources generally limited the extent of the acquisition and assembling of environmental knowledge, but we did not observe other kinds of restrictions related to the funding.

Other research has emphasized the vitality of scientific knowledge to the environmental expertise of ENGOs. For example, the organizations studied by Eden et al. (2006) often referred to scientific sources as preeminent authorities in environmental debates. Or, Yearley (2017: 11) writes: ‘Environmentalists … are obliged to act as communicators of science and technology because empirical claims about the state of the natural environment are core to their message.’ This suggests that ENGOs are what Guston (2001) calls boundary organizations, working at the border between ‘the two relatively different social worlds of politics and science’ (Guston, 2001: 401). From this perspective, ENGO actors would be expected to engage in knowledge brokering, facilitating the communication between scientists and policymakers in the environmental area.

No doubt, scientific knowledge and interaction with scientists were important to our interviewees. However, their accounts generally presented scientific knowledge as ubiquitous and opaque. The interviewees did not consider that they brokered particular instances of scientific knowledge, and they did not report much direct contact with scientists. Rather, they articulated a more general appraisal of a wider set of knowledge and expertise as necessary assets in their work.

Expertise is essential, right. At least the green movement in Norway and in the Nordic countries, they are in a way so nerdy, ‘knowledge-nerdy’. … We live in a kind of paradigm where expertise, not being wrong – being precise – is mandatory for being allowed to enter to the table [of policy exchanges]. (B1)^2^

This point of view was shared across the organizations, independent of their thematic preferences and practices. While some of the ENGOs had several scientists as members, the interviewees did not talk much about these members or other scientists disseminating knowledge to them. Rather, they tended to describe the interaction with science and scientists as indirect. Moreover, they emphasized that they also used many other sources when they compiled the knowledge they needed in a particular case (see also Cash et al., 2003; Eden et al., 2006). In this manner, the interviewees argued that they were the main active parties in their acquisition and assembling of environmental knowledge, which usually were driven by demand. They searched for knowledge when needed in their engagement with a particular concern, a controversial construction project, a public hearing or a policy proposal.

The searches were described as pragmatic, using a wide range of sources. They were frequently referred to as projects to identify and organize the concrete acquisition and assemblage of the knowledge they needed. Such projects were not the outcome of advance planning or formatted by standard procedures. The individual ENGO employee decided case by case how to proceed and the interviewees told that their strategies for gathering knowledge were exploratory, to some extent idiosyncratic and embedded in networking. ‘[Knowledge acquisition] is a somewhat stone-by-stone project, to connect with actors that might be relevant. We work with very different partners, like [Company X], [Company Y], and [Municipality Z], if you understand’ (E2).

It seems misleading, then, to characterize the ENGOs as boundary organizations. We found no clear indications that they occupied the expected intermediate position in a chain of knowledge displacements, making the interviewees primarily engage in knowledge brokering. Their accounts suggested more complex practices where ENGO actors played an active role in acquiring, interpreting and assembling environmental knowledge from a multitude of sources. Science was important, but in a diffuse manner. Therefore, we needed different concepts to make sense of the learning processes of the ENGOs.

## Mediation and rhizomic learning

Innovation studies (e.g., Lundvall, 2016) and knowledge management (e.g., Choo, 2016) have long been concerned with learning and acquisition of knowledge through search procedures. Still, these contributions are less useful to us because they analyze processes related to economic achievements and in more resource-rich contexts than those of the ENGOs. In science and technology studies, there are relevant concepts and approaches unrelated to the boundary organization idea. For example, we could follow Nowotny et al. (2003) in arguing that the ENGOs demand-driven knowledge acquisition and assemblage exemplifies ‘problem-solving in the context of application’ and thus a transdisciplinary approach where ENGO actors combine a diversity of science-based and experience-based knowledge. Jasanoff (1997) makes a related argument, emphasizing how such organizations may gain access to domains of localized experience and knowledge from the grass roots.

However, these approaches offer less insight into the processes of knowledge acquisition, knowledge assemblage and learning that concerns us. They do not sufficiently engage with the complexity that we observed from our interviewees’ accounts (see also Eden et al., 2006; Fähnrich, 2018). Alternatively, we may turn to Latour (2005), who argues that the movement of knowledge should be expected to involve change, through acts of mediation. These acts involve the work of, for instance, ENGO actors identifying and eventually transforming, translating and modifying knowledge. When they prepare to communicate with policymakers in particular decision-making contexts by acquiring and assembling knowledge, we expect them to adapt this knowledge to make it relevant and to appear as trustworthy. However, mediation is a broad concept that, above all, highlights the malleability of circulating knowledge, which is not always a relevant focus.

We introduce the concept of rhizomic learning to describe the ENGO actors’ acquisition and assemblage of environmental knowledge. We believe that this concept better captures the purposefulness as well as the multiplicity of sources and the complexity of the knowledge gaining processes of the ENGOs. Above all, we see the concept as providing a better understanding of the diffuse but still vital role of science in the knowledge practices of these organizations. We return to this issue repeatedly throughout the paper.

We consider ENGO actors’ acquisition and assembling of knowledge to be learning because this aptly catches their explorative and experience informed approach. We call the process rhizomic to emphasize the non-linear, interactive and contingent features that we observed from the interviews. The concept draws on the work of Deleuze and Guattari (1987), who introduced the rhizome as a metaphor to capture the non-linear and non-hierarchical features of knowledge. It has been picked up by STS scholars such as Martin (1998: 31), who claims that it is useful in empirical studies since it captures the fractured, intermittent relationships between science (knowledge) and the rest of the culture. Similarly, Latour (1999) links ‘rhizome’ to actor-network theory (ANT), emphasizing that it signifies the series of transformations occurring through mobility of knowledge. Still, Martin’s and Latour’s ideas remain abstract. In this paper, we want to explore empirically what rhizomic learning means. When we characterize the knowledge practices of ENGO actors as rhizomic learning, happening in a purposeful context of environmental politics, what are main features of these processes? How does this illuminate the relationship between ENGOs and science?

We observed that the ENGO actors engaged in rhizomic learning by situating themselves in flows, from various sources, of environmental knowledge, assembling and transforming these flows into concrete, policy relevant knowledge claims about the environment and how environmental issues should be addressed. They connected different pieces of knowledge into heterogeneous statements that included value assessments and suggestion of actions. ‘[We try] to influence the perception of reality. Because efforts to influence bureaucrats are about the perception of fact or, often, interpretation of what the facts mean, or eventually, indicating what possible instruments to suggest’ (E4).

To understand the practice of rhizomic learning, our strategy was to analyze the interviewees’ accounts of how they identified and related to different sources of knowledge. We were told about four main sources or activities: (1) reading papers and reports, (2) networking and direct communication with relevant people, (3) internet searches, and (4) their own and colleagues’ education and experience. This list of sources is not very surprising, although the ways in which the sources were combined and assembled gave evidence of rhizomic connections and transformation. More telling details about the rhizomic properties of the learning process of the ENGO actors became evident from how they described and reflected about the activities in which they were engaged.

## Rhizomic learning through assembling and assessing sources of environmental knowledge

### Source 1: Reading

Unsurprisingly, reading was an important part of the acquisition and assembling of environmental knowledge, and it was usually initiated through a project where an ENGO actor needed more knowledge. The most common motivation to search for knowledge through reading was to obtain specialized knowledge, relevant to a current concern, whenever needed. The interviewees told that they seldom had the opportunity to study a text just to get a professional update. Rather, the acquisition of new knowledge was hurried.

I’ve got a list of things I wish I could read up on, kind of, but I very rarely find the time to do so. So, the result is that I do it [read up] whenever I need to … sometimes you need to send out a press release within an hour and have to collect the [required] knowledge; and something you have from before. (F2)

We asked about what kind of documents the interviewees read, how they identified items to read and about the process of reading. Interviewees said they seldom based their learning about an issue on only a single text, and usually the process of assembling knowledge could be described as an intertextual practice. Clearly, the policy context and the purpose of the reading involved interpretation and thus mediation. Rhizomic features were also apparent in accounts of how the interviewees related to science. As noted, direct communication with scientists was less frequent than anticipated from previous research. Nevertheless, we expected scientific papers to be important sources of knowledge in the learning processes of the ENGO actors. However, when we asked interviewees about what they read, such documents were rarely mentioned. Only a couple of the interviewees said they found peer-reviewed scientific papers to be vital because of their perceived reliability as sources of expert knowledge. Rather, a common and often prompt response to the question of what they read was: ‘reports’.

When asked about further details, the interviewees tended to be more hesitant and staggering, chiefly stating that their reading could be documents of any kind, from several varieties of sources. When pressed for examples, several mentioned reports from the Intergovernmental Panel on Climate Change (IPCC) and from research institutions. However, only a few of the interviewees who mentioned the latter were able to exemplify what this might entail. Again, they became vague in their answers, using phrases like ‘that depends’ and ‘it varies’, or they extended their answer to include reports from ministries, local governments and other similar sources, including their own organization. We see this as reflecting the rhizomic quality of their learning in the sense that their acquisition and assembling of knowledge appeared to be messy and opaque. Moreover, science-based knowledge could come from a multitude of sources, mediated by other actors, and without reference to a definite origin.

At the same time, the interviewees pointed out that it was important to be critical, to look not only for knowledge and information that supported their own views. Being caught doing the latter would not serve their case: ‘There is no doubt that when we read, we also do this with a critical eye; exactly because we don’t want to be accused of promoting somebody else’s work without having thought of the fact that it only supports our case’ (C1). A few acknowledged that, given the huge amount of information available, there was always the risk of being biased when choosing what information to trust. Nevertheless, they maintained that the ability to assess the quality of particular pieces of environmental knowledge was one of their core competences: ‘We are, just like everybody else, I believe, running the risk of using the knowledge that best fits our system of beliefs, kind of. That’s something towards which we always have to be sensitive’ (A1). Thus, rhizomic learning can involve informal assessment of validity, not relying merely on peer review and other formal assessments.

Scientific and trade journals were not at the top of the list of what the ENGO employees said they read to acquire knowledge, and neither were such sources as news media, newsletters and digital media like blogs and twitter messages. However, the interviewees mentioned academic databases as important sources, ResearchGate in particular, but they described the usefulness of these sources to knowledge in contradictory ways. It appeared that most of the interviewees dealt with these sources rhizomically in the sense that they used them quite unsystematically.

To summarize, gathering information and learning through reading printed documents was problem-driven and frequently shaped by tight time constraints. This could limit the scope of the ENGO actors’ search for sources, constrain their learning and consequently reduce the extent and amount of rhizomic flows into their acquisition and assembling of environmental knowledge. On the other hand, according to the interviewees, written documents were not their most important sources of knowledge. Almost without exception, the interviewees expressed that they usually preferred other sources. In particular, they favored oral input gained by contacting people, both colleagues and external sources, assumed to possess the knowledge they needed.

### Source 2: Networking and verbal communication

All interviewees used talking to other people to access knowledge and expertise, and this provided a strong oral feature to their acquisition work. Again, they found it difficult to provide clear, plain and unambiguous descriptions of how such communication was initiated, developed and transpired. The interviewees did not take a systematic approach with respect to this way of acquiring environmental knowledge either. Rather, their practice appeared incidental and contingent on whomever the ENGO representative knew, knew of, had any kind of personal link to, or simply happened to meet. ‘It is often people you have met a few times’ (E2). Interviewees mentioned the internal movement of knowledge within their organization as useful and vitally important, but this also tended to happen accidentally. ‘[It] can involve anything from having meetings, informal chats during lunch, but it’s just as much going with that person to different – yes, external meetings and other encounters like that’ (F2).

When asked for further details or examples, interviewees mentioned that they knew university professors or researchers whom they could call if needed or employees from organizations that they had worked with before. Many also had contacts within industry that they could approach. Communication, of varying degree of formality, took place by phone, via email, through meetings or simply when somebody relevant happened to be in the vicinity. In general, though, acquaintances were not contacted on a regular basis or in formalized ways. Instead, also such encounters were based on case-specific needs. The ENGO actors described their sources of verbal knowledge by referring to their more or less rhizomic networks, usually approached in incidental and contingent ways. ‘It might be … I know people at [Research Institute X], you know. [Competence Centre Y], that deal with the kind, anything from research to kind of applied use of knowledge, that know of … maybe other research results’ (F3).

Scientific knowledge seems to be seen as ubiquitous and accessible in complex and composite ways, not as something that actually flowed linearly to the ENGOs. One of the interviewees also pointed out that with respect to certain organizations, especially within industry, it was important to know somebody to get access to their information or knowledge. On the other hand, getting to know practically anybody in Norway appeared to be unproblematic to the interviewees. Almost without exception, they described the people with whom they communicated as quite enthusiastic about providing information to ENGOs. The interviewees experienced themselves as being seen as actors with good intentions, not constituting a threat to the parties with whom they cooperated. In particular, those with previous experience from industry seemed surprised but also enthusiastic about the benevolence of the people they approached and who even might approach the ENGO on their own initiative to supply them with information.

[This experience] is also something that’s quite unique compared to when I worked as a consultant for instance; it’s how much good information that just keeps falling into our laps. When we were consultants, we sat there searching, reading reports. Now, it’s like if you go to a meeting with [State enterprise X], the CEO greets you, and well, they give you their core information and estimates, kind of, and you get so much information made so easily available. The research department of [State enterprise Y] is kind of – is considered one of the best in the country, and not even the Ministry of Petroleum and Energy, kind of, has free access to [their data]. Moreover, we receive so nicely quantified, extrapolative scenarios and that kind of stuff. Incredibly useful! (A2)

In line with the idea of rhizomic learning, interviewees described their acquisition and assembling of environmental knowledge as frequently depending upon luck and coincidence. On the other hand, many of them also stressed the importance of their networks. Clearly, network-building was an intentional, strategic part of their job. Some even said that their organizations used labor turnover as a deliberate network-building strategy. In this manner, the ENGO was able to utilize a broad spectrum of human resources, with diverse forms of education, experience and contacts. The interviewees who told of such a strategy primarily accentuated the movement of people among political parties, relevant parts of public administration, and ENGOs. Many also mentioned the loyalty they experienced from former employees, who were willing to share their professional knowledge, expertise and experience. The interviewees further reported that they interacted with civil servants. However, these actors were rarely presented as pivotal sources of knowledge, but rather as partners in factual exchanges that could take place, for instance, during board meetings, conferences or seminars where both parties were represented.

ENGO employees commonly suggested that the number of people involved in environmental policymaking in Norway is quite limited. We were told that the people involved in such affairs kept running into each other at different venues and meetings. This meant that they often were exposed to the same news, information and knowledge. As a result, several of the interviewees argued that the people involved in these exchanges chiefly were in agreement with respect to the quality and reliability of the environmental knowledge they discussed. This situation also meant that, normally, it was just a matter of time when new insights into relevant issues would be shared by everyone engaged in the environmental policy field in Norway.

We see this communal approach to sharing and assessing knowledge as an important ingredient of rhizomic learning. The interviewees described a network-based traffic in environmental knowledge, where information and insights could have many roots and result from distributed knowledge practices. This has several interesting aspects. First, the rhizomic features of the communal practice meant that it often was difficult to trace knowledge to a single source, which was not considered a problem. Second, the community involved in environmental policymaking interacted in ways that we interpret as a process of collective assessment of the quality of new knowledge, including its policy relevance. In this context, rhizomic learning meant addressing a complex of sources but also a shared engagement with sense-making of environmental knowledge among relevant actors. Third, the rhizomic qualities of the ENGO-based appropriation of knowledge made the outcome of the process less predictable, since access to people with relevant insights might not be possible within the timeframe of the relevant project. Digital media could be helpful in overcoming this latter challenge.

### Source 3: Digital media

The interviewees mentioned digital media, especially the internet, as a main vehicle of the rapid and comprehensive spread of knowledge throughout the environmental policymaking community. How was this used? All the interviewed ENGO employees confirmed that they used digital media, in particular internet search engines, to acquire relevant knowledge and information. To what extent and in what way varied. Some of the interviewees remarked that to ask whether they Googled was silly because the answer was so obvious. Whenever convenient, they might even perform ‘open’ searches for relevant information to update themselves.

Well, it’s not uncommon to use Google, you know, to put it like that. Searching for stuff, to look if something comes up that – either a good article or reports or well, something like that. … Yes, also if there are discussions about environmental consequences of things, well, then it’s often to look up life-cycle analyses. … I would certainly say that if I am uncertain about something, it often becomes like – search online! (F3)

Others were more reluctant to admit that they used the internet to acquire environmental knowledge. They would emphasize that they were always very critical of what they read on digital media. These interviewees were also careful to stress that they only used Google to find concrete pieces of information that they already knew existed, such as data that they needed to look more into, or that they wanted to read to refresh their mind, but that had been acknowledged as valid through other channels. ‘Eh well, [I] am a bit skeptical towards just blind Googling. Then it must be at a very early stage of something. I rarely think that I’m working at a very early stage of something. There is always something you can find a bit about through the network [of people], I think’ (A2).

Interviewees typically expressed skepticism towards accepting information found online, unless it could be assessed also through other sources. On the other hand, they seemed to feel quite confident that they were able to do such assessments themselves even if they found it difficult to exemplify how they did this. Often, they would just refer in a general way to ‘contacts’. Surprisingly, only a couple of the interviewees mentioned their educational background as a resource of assessing the quality of the information they found on internet. To what other ends did they consider their formal education useful?

### Source 4: Education-based expertise in their own organization

Most of the ENGO interviewees held master’s degrees, some even PhDs. Their educational background was diverse, covering fields like nature and resource management, biology, geography, environmental politics and regulations, architecture, policy studies and sociology. Presumably, they held expertise relevant to their appropriation of environmental knowledge. How was this expertise employed?

In general, the interviewees agreed that in their line of work, they needed a considerable amount of environmental expertise to carry out their job. There were, as expected, those who deemed their educational background as necessary, or at least very useful, to make them able to carry out their work in a satisfactory manner. Nevertheless, many shared the opinion that no single educational background provided the kind of expertise they needed to succeed in their work. Other key skills were required as well, and the interviewees emphasized the need to assemble knowledge from different disciplines.

Now, we have a specialist department, which is made up of quite a few people who have … academic qualifications, right? Thus, there are master’s students in renewable energy, people with a background in law, engineers, and so on … and that is important, but this is not the only thing needed to succeed with what we want to achieve. Thus, the professional understanding, the ability to understand problem complexes from having a professional background is increasingly necessary to be able to succeed with some of the things that we are doing. Nevertheless, you have to have a talent in thinking holistically, generally, and the ability to extract the essence of already existing knowledge, right? (A1)

Other interviewees valued their educational background differently in relation to their current work. They expressed that their education probably made a difference, making them better at their job. However, they considered their education to be more of a backdrop for their efforts of acquiring and assembling environmental knowledge, arguing that such specialist competence was not required or vital to their ability to perform their professional work. A common narrative of the interviewees was that whatever background they had, with a degree in social science, economics, natural science or merely organizational experience, in practice, the important part was their personal skills and ability to manage the tasks they faced in acquiring and assembling environmental knowledge. Hence, being a generalist was just as useful as any other background to engage in rhizomic learning.

The most surprising finding from this part of the interviews was how frequently some interviewees claimed that specialist knowledge obtained through formal education made little or no difference whatsoever to their ability to adequately perform their everyday tasks. For these interviewees, the core skill for people like them was the ability to keep up to date regarding the issues with which they were dealing. ‘You don’t have to be an economist to do these calculations [that I do]. They are only about being able to calculate … . Expertise is having followed a field for a while’ (E1).

Furthermore, some interviewees expressed that what really mattered to succeed in keeping up to date was enthusiasm and ‘hands-on experience’. For example, they could emphasize engagement in ENGO activism as a primary road to successful achievements. They even explicitly said that if they had to choose between distinct types of knowledge, they would prefer experience-based knowledge – acquired from having worked within their organization for a while – over knowledge acquired through education. Nevertheless, it was more common to express the need to be able to combine a variety of knowledges.

The moderate appreciation of their educational background suggests that the ENGO actors did not use this source to access knowledge in a linear fashion but rather as a rhizomic learning competence. When they appreciated method skills and analytic training, they saw this as helpful to navigate the multiplicity of sources they used as well as their management of the sense-making activities and assessment of validity of knowledge claims involved in assembling of environmental knowledge.

## Conclusion: Features of rhizomic learning

Previous research suggests that ENGOs depend on science as an authoritative source of environmental knowledge (e.g., Yearley, 2017). We do not dispute this claim, but our study suggests that ENGOs mainly learn about scientific findings in a complex, distributed, non-linear and mediated manner. Moreover, in the process of assembling environmental knowledge, science is combined with experience, political savoir faire and value assessments. We describe this as rhizomic learning, which provides an alternative understanding of science communication compared to the common focus on scientists as more or less troubled communicators of science (e.g., Davies and Horst, 2016). Usually, the ENGO actors did not directly assess and acquire science. For example, few of our interviewees read scientific papers. Rather, science was described as ubiquitous, as available through a multitude of voices and connections. Typically, the ENGO actors found it difficult to specify how they engaged with scientific sources while still emphasizing the importance of scientific knowledge.

We have discussed rhizomic learning based on the interviewees’ accounts of how they engaged with their four main sources of environmental knowledge; written material, networks, digital media and educational background. To conclude, we want to highlight five key features to clarify rhizomic learning as an empirical concept, based on our observations. First, rhizomic learning meant that the interviewees engaged in contingent ways with a complex diversity of sources. From their accounts, it was clear that they understood environmental knowledge as distributed across a multitude of loosely connected actors, nodes and institutions. The interviewees needed to navigate this multitude. Networking was a main strategy to engage with the complex flows of environmental knowledge and thus a vital ingredient of the rhizomic learning practices.

Second, rhizomic learning is characterized by pragmatism. The interviewees emphasized their pragmatic engagement with environmental knowledge, which also reflected an unsystematic relationship with scientific knowledge, a lack of standardized approaches to the acquisition of knowledge, and a practice of learning that was problem-based and needs driven. This pragmatism contributed to the complexity of acquiring and assembling knowledge because the choice of and approach to sources of knowledge were contingent on the actual context of the learning.

A third feature of rhizomic learning is the opacity of the process of acquiring and assembling knowledge. Generally, the interviewees found it difficult to provide concrete answers when they were asked about the sources they used and their strategies of acquiring the knowledge they needed. The opacity of rhizomic learning was clearly co-produced with the two characteristics mentioned above, not the least the pragmatism of the ENGO actors in their engagement with environmental knowledge.

Fourth, we observed outspoken communal qualities in the interviewees’ accounts, qualities that shaped their rhizomic learning. As noted, they told about their engagement with a diversity of sources of knowledge. However, at the same time, they emphasized that environmental knowledge was distributed across a community that provided multiple ways of acquiring insights as well as a validation of knowledge claims. The community of environmental policy actors was more important to establish trust in knowledge than scientific peer review. Thus, rhizomic learning appeared to be situated in this community in a fundamental manner, which shaped the assessment practices.

Finally, and in line with our initial argument, mediation was an integrated feature of rhizomic learning, meaning that knowledge tended to be translated or adapted to the problem context. When the interviewees said it was important to combine sources, this clearly meant that they engaged in situated forms of interpretation and synthesis also to make sure that the knowledge they would supply, for example, to policymakers, was considered relevant and reliable.

Most of the interviewees agreed that relevant environmental knowledge was, in principle, available to everybody engaged in the environmental field at any time. Thus, merely providing knowledge could not be what made ENGOs interesting to their partners. Rather, we have observed that it was their capacity of rhizomic learning – not the least the purposeful mediation and assembly of knowledge from a multitude of sources – that the ENGO actors expected to make them appear as competent, relevant and reliable. Their extensive networking activities were an important part of this, helping them to achieve a good overview of possible partners and accessible sources of knowledge. In addition, they learned the rules of the game, such as the policy culture of government. This facilitated their moving within and between public administration and policymakers as well as science and other providers of environmental knowledge.

The concept of rhizomic learning highlights how the acquisition and assembling of knowledge may be shaped by interactions within a particular community, involve a multitude of sources, be opaque and contingent. We believe that this concept may be fruitfully applied in other contexts such as other kinds of policymaking, engineers’ problem-solving, or consulting, to provide new insights into the non-linear qualities of the related processes of assembling and making knowledge.

## Notes

1. In 2018, environmental organizations in Norway had a total income of 314 million NOK (US$ 35 million), 20 per cent – or 64 million (US$ 7 million) from industrial actors (Hinna, 2019). The rest came from government subsidies and membership or associate membership fees, including private donations. The economic resources of the ENGOs are not insignificant but require economic sobriety on the part of the organizations.

2. The organizations are referred to by letters (A to H), and the interviewees from the same organization by numbers. Thus, when we quote E2, we quote interviewee 2 from ENGO E. In this way, we distinguish the interviewees and allow readers to see if they belong to the same organisation or not.
